# Supplementary material for: Loss of PHF8 induces a viral mimicry response by activating endogenous retrotransposons
Source: Nat Commun. 2023 Jul 15;14:4225. doi: 10.1038/s41467-023-39943-y (PMC10349869; doi:10.1038/s41467-023-39943-y)
Supplement: Supplementary file 1 — Supplementary Information [file 41467_2023_39943_MOESM1_ESM.pdf]

Supplementary Information for

Loss of PHF8 induces a viral mimicry response by activating endogenous retrotransposons

Yanan Liu[1,5], Longmiao Hu[1,5], Zhengzhen Wu[1,5], Kun Yuan[1], Guangliang Hong[2], Zhengke Lian[1], Juanjuan Feng[1], Na Li[1], Dali Li[1], Jiemin Wong[1], Jiekai Chen[3], Mingyao Liu[1], Jiangping He[2,*], Xiufeng Pang[1,*]

[1]Shanghai Key Laboratory of Regulatory Biology and School of Life Sciences, East China Normal University, Shanghai 200241, China; [2]Guangzhou Laboratory, Guangzhou 510005, China; [3]Key Laboratory of Regenerative Biology of the Chinese Academy of Sciences and Guangdong Provincial Key Laboratory of Stem Cell and Regenerative Medicine, Guangzhou Institutes of Biomedicine and Health, Chinese Academy of Sciences, 510530 Guangzhou, China.

[5]These authors contributed equally to this work.

To whom correspondence should be addressed:
Dr. Xiufeng Pang
School of Life Sciences
East China Normal University
500 Dongchuan Rd.
Shanghai 200241, China
Office phone: +86-21-24206942
Office fax: +86-21-54344922
E-mail: xfpang@bio.ecnu.edu.cn

Dr. Jiangping He
Guangzhou Laboratory
190 Kaiyuan Rd.
Guangzhou 510320, China
Office phone: +86-020-62726076
Email: he_jiangping@gzlab.ac.cn

The PDF file includes:
Supplementary text
Supplementary figures 1 to 7

Supplementary Figure 1
Related to Figure 1

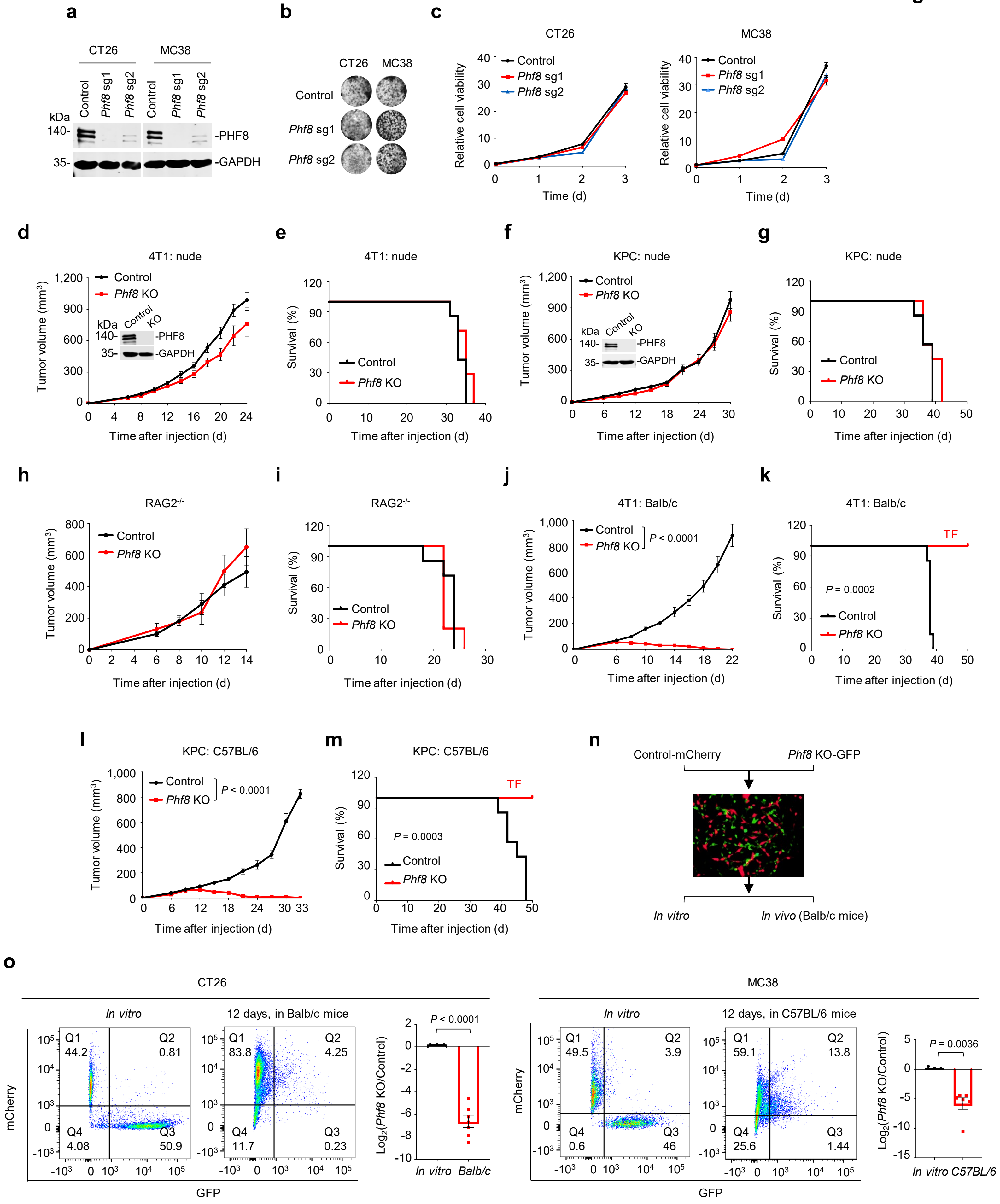

Supplementary Figure 1
Related to Figure 1

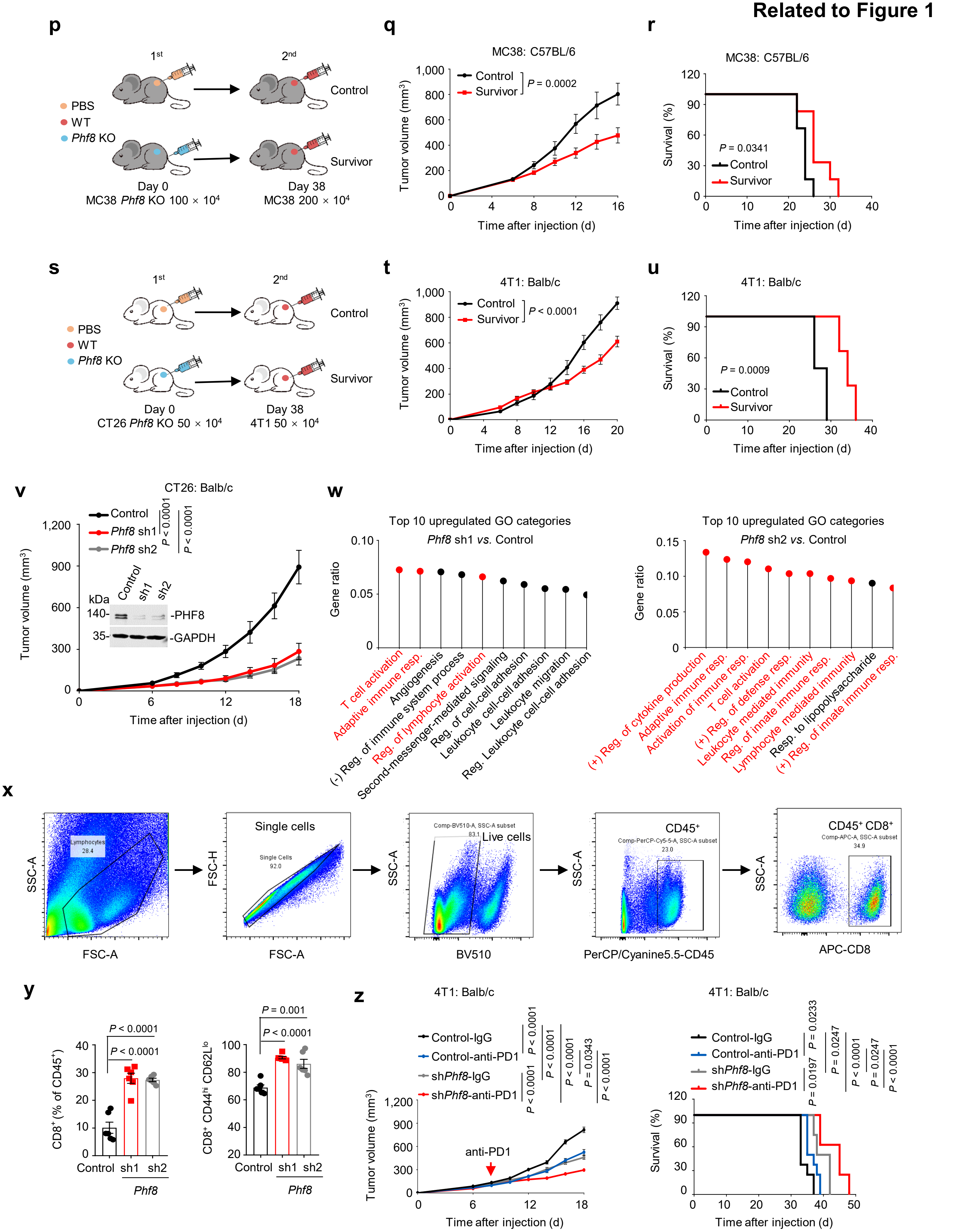

Supplementary Figure 1 | The effects of *Phf8* knockout on tumor cell proliferation *in vitro* and tumor growth *in vivo*. a, Western blot analysis of PHF8 expression in *Phf8* wild-type (the vector control) and *Phf8* knockout (KO) murine tumor cells induced by two independent *Phf8* sgRNAs**. b,** Representative images of clonogenic growth of the vector control and *Phf8* KO tumor cells. **c,** Cell viability curves of *Phf8* KO CT26 and MC38, measured by the sulforhodamine B colorimetric methods at various time points. Relative cell viability was calculated by setting the control group as 1. Values are expressed as mean ± SEM. *n* = 3 biologically independent samples. **d-g**, Tumor growth curves (**d**, **f**) and Kaplan-Meier survival curves (**e**, **g**) of *Phf8* KO 4T1 and KPC cells and their corresponding vector control cells. In each cases, about 250,000 (4T1) or 100,000,0 (KPC) tumor cells were inoculated subcutaneously into nude mice, and tumor formation was monitored. *n* = 7 tumors per group. Western blot analysis of PHF8 expression in *Phf8* wild-type and knockout murine tumor cells (4T1 and KPC). **h, i,** Tumor formation of the vector control and *Phf8* KO MC38 cells (**h**), and Kaplan-Meier survival curves (**I**) of the host mice. About 1,000,000 tumor cells were inoculated subcutaneously into RAG2$^{-/-}$ mice. *n* = 7 and *n* = 5 for the vector control and *Phf8* KO group, respectively. **j-m**, Tumor growth curves (**j**, **l**) and Kaplan-Meier survival curves (**k**, **m**) of the vector control and *Phf8* KO 4T1 and KPC cells. In each cases, about 250,000 (4T1) or 100,000,0 (KPC) tumor cells were inoculated subcutaneously into Balb/c (4T1) or C57BL/6 (KPC) mice. Tumor formation was monitored. *n* = 7 tumors per group. **n,** Details of the *in vivo* competition assay. **o,** Changes in the ratio of mixed control-mCherry and *Phf8* KO-GFP CT26 cells (*left*) or MC38 cells (*right*) after 12-day growth in Balb/c or C57BL/6 mice, as determined by flow cytometry analysis. And quantitative data is shown. *n* = 3 and *n* = 7 (CT26) or *n* = 6 (MC38) biologically independent tumor samples for *in vitro* and *in vivo* assays, respectively. **p-r**, Treatment scheme (**p**), tumor growth curves (**q**) and Kaplan-Meier survival curves (**r**) of host mice after rechallenge with 2,000,000 *Phf8* wild-type MC38 tumor cells in C57BL/6 mice that remained tumor-free for 38 days after initial challenge with 1,000,000 *Phf8* KO MC38 cells. The control group referred to tumor-naive C57BL/6 mice challenged with *Phf8* wild-type MC38 cells. *n* = 6 tumors per group. **s-u**, Treatment scheme (**s**), tumor growth curves (**t**) and Kaplan-Meier survival curves (**u**) of host mice after rechallenge with 500,000 *Phf8* wild-type 4T1 tumor cells in Balb/c mice that remained tumor-free for 38 days after initial challenge with 500,000 *Phf8* KO CT26 cells. The control referred to tumor-naive Balb/c mice challenged with *Phf8* wild-type 4T1 cells. *n* = 6 tumors per group. **v,** Tumor formation of the vector control and sh*Phf8* CT26 tumors. About 500,000 tumor cells were inoculated subcutaneously into Balb/c mice, and tumor formation was monitored accordingly. *n* = 8 tumors per group. Western blot analysis of PHF8 expression in *Phf8* wild-type and knockdown CT26 cells (*inset*). **w,** Gene ontology (GO) analysis by comparing RNA-seq data from *Phf8* sh1 (*left*) or *Phf8* sh2 (*right*) CT26 tumors with the vector control tumors. Shown are selected top 10 upregulated GO terms (FDR < 0.001). **x,** Representative flow-cytometry gating strategy for quantifying the numbers of various immune effector cell subsets in murine tumors. **y,** Quantitative estimate of various immune effector cells in the vector control and sh*Phf8* CT26 tumors, as analyzed by flow cytometry. Cell populations were identified as CD8^{+} T cells (CD45^{+} CD8^{+}), CD44^{hi} CD62L^{lo} CD8^{+} T cells. *n* = 6 tumors per group. **z,** Tumor growth curves (*left*) and Kaplan-Meier survival curves (*right*) of Balb/c mice inoculated with 250,000 *Phf8* wild-type and knockdown 4T1 cells. Xenograft tumors were treated with IgG control or anti-PD-1 antibodies 8 days after tumor cells inoculation. *n* = 8 mice per group. Data are presented as the mean ± s.e.m.. TF, tumor free. Unpaired two-sided Student's *t*-test in **o**, two-way ANOVA in **d**, **f**, **j**, **l**, **q**, **t**, **v** and **z** (*left*), log-rank test in **k, m, r, u** and **z** (*right*). The immunoblots in **a**, **d**, **f,** and **v** are representative of three independent experiments. Source data are provided as a Source Data file.

Supplementary Figure 2
Related to Figure 2

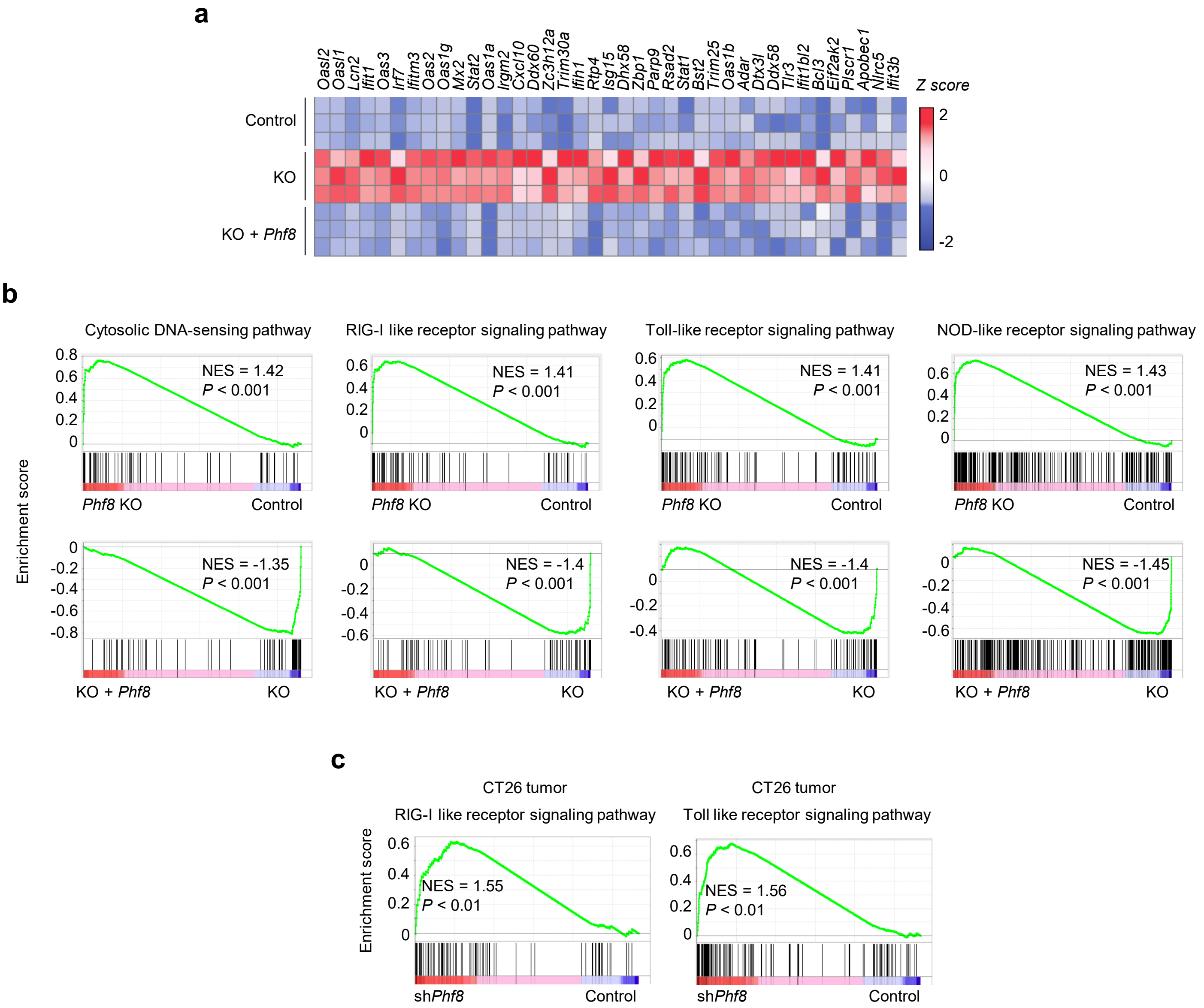

Supplementary Figure 2 | *PHF8* depletion activates endogenous nucleic acid sensing pathways. **a,** Heatmap showing differential expression (P < 0.05) of antiviral defense-related genes in the control, *Phf8* KO and *Phf8 KO* + *Phf8* CT26 cells. Upregulated genes (Log_2 FD >1, P < 0.05) in *Phf8* KO CT26 cells compared with the vector control cells and downregulated genes (Log_2 FD < -1, P < 0.05) in *Phf8* KO + *Phf8* CT26 cells compared with *Phf8* KO CT26 cells are shown. n = 3. P value was calculated using DESeq2 package (see details in "Methods"). **b,** Gene set enrichment analysis of RNA-seq data showing cytosolic RNA-sensing and DNA-sensing pathways that were upregulated in *Phf8* KO CT26 cells compared with the vector control cells (*upper*) and corresponding pathways downregulated in *Phf8* KO + *Phf8* CT26 cells compared with *Phf8* KO cells (*lower*). n = 3, NES, normalized enrichment score. **c,** Gene set enrichment analysis of RNA-seq data showing cytosolic RNA-sensing pathways that were upregulated in sh*Phf8* CT26 tumors compared with the vector control tumors. n = 8. P values in **b** and **c** using a two-sided Kolmogorov–Smirnov test.

Supplementary Figure 3
Related to Figure 3

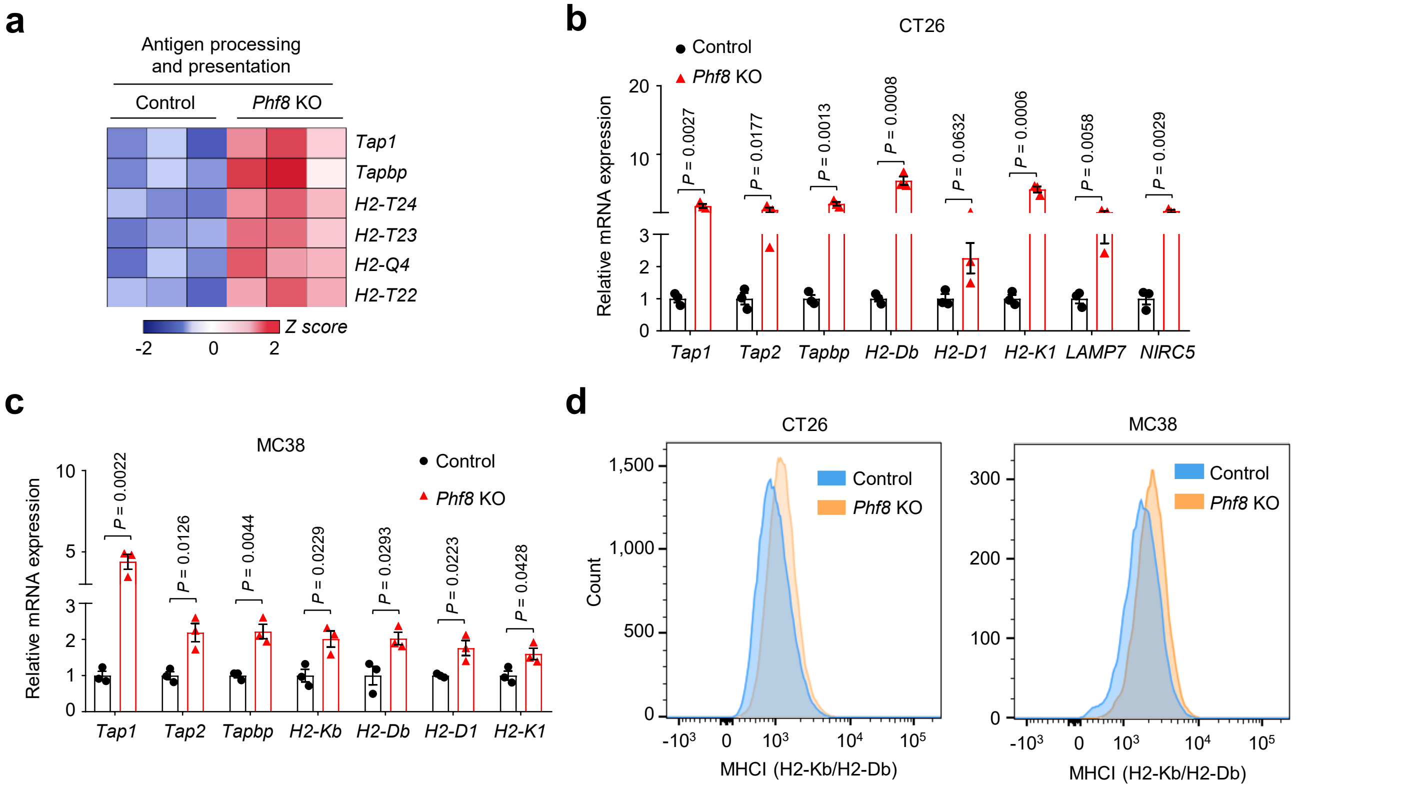

Supplementary Figure 3 | *PHF8* depletion enhances antigen processing and presentation. a, Heatmaps showing induction of antigen processing and presentation-related genes ($P < 0.05$) in the vector control and *Phf8* KO CT26 cells. $n = 3$. P value was calculated using DESeq2 package (see details in "Methods"). **b, c,** RT-qPCR analysis of transcripts of antigen processing and presentation genes in CT26 (**b**) and MC38 cells (**c**). **d,** Flow cytometry analysis showing cell surface expression of MHCI levels in the control and *Phf8* KO CT26 (*left*) and MC38 cells (*right*). For **b** and **c**, values are expressed as mean ± SEM. $n = 3$ biologically independent samples. Unpaired two-sided Student's *t*-test.

Supplementary Figure 4
Related to Figure 4

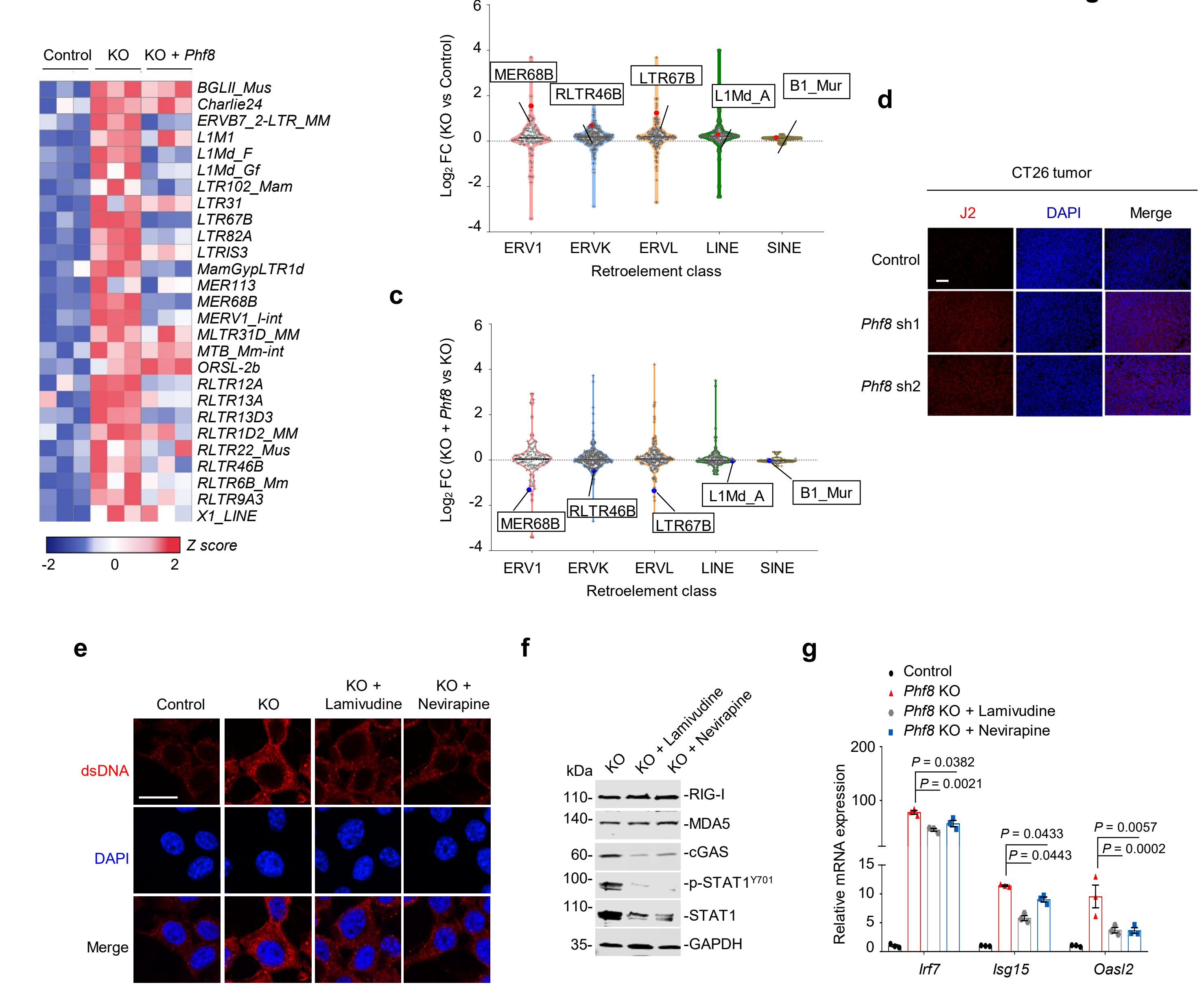

Supplementary Figure 4 | *PHF8* regulates endogenous retrotransposons expression. a, Heatmap showing differential expression (P < 0.05) of retrotransposons in the control, *Phf8* KO and *Phf8* KO + *Phf8* CT26 cells. Upregulated retrotransposons (Log_2 FD >1, P < 0.05) in *Phf8* KO CT26 cells compared with the vector control cells and downregulated retrotransposons (Log_2 FD < -1, P < 0.05) in *Phf8* KO + *Phf8* CT26 cells compared with *Phf8* KO CT26 cells are shown. n = 3. P value was calculated using DESeq2 package (see details in "Methods"). **b, c,** the classes of PHF8-regulated retrotransposon. The differentially expressed retrotransposon classes by comparing *Phf8* KO CT26 with the vector control cells (**b**) and the differentially expressed retrotransposon classes by comparing *Phf8* KO + *Phf8* CT26 cells with *Phf8* KO cells (**c**). n = 3. **d,** Immunofluorescence staining of dsRNA for CT26 harvested 18 days after tumor cell injection. Representative images of 3 independent experiments. Scale bar, 50 μm. **e,** Immunofluorescence staining of dsDNA (red) and DAPI (blue) in CT26 cells. CT26 control or *Phf8* KO cells were treated with 30 μM Lamivudine or 30 μM Nevirapine for 48 hours before staining. Representative images of 3 independent experiments. Scale bar, 20 μm. **f, g,** Western blot (**f**) and RT-qPCR (**g**) analysis of *Phf8* KO CT26 cells treated with 30 μM lamivudine or nevirapine for 48 hours. Values are expressed as mean ± SEM. n = 3 biologically independent samples. Unpaired two-sided Student's t-test. The immunoblots in **f** are representative of three independent experiments. Source data are provided as a Source Data file.

Supplementary Figure 5
Related to Figure 5

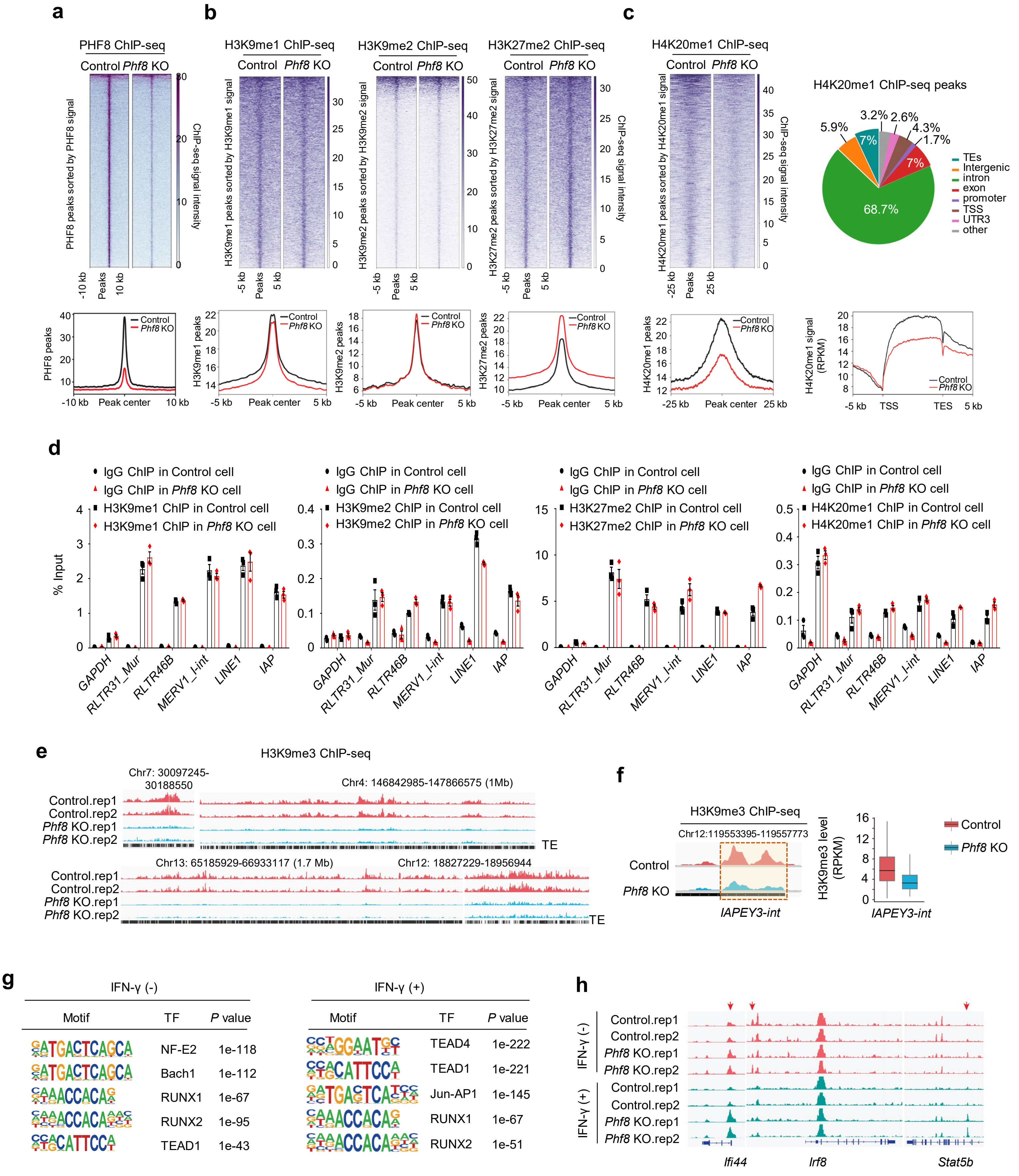

g

IFN-γ (-)

Motif	TF	P value
GATGACTCAGCA	NF-E2	1e-118
GATGACTCAGCA	Bach1	1e-112
CAAACCACAG	RUNX1	1e-67
CAAACCACAAGC	RUNX2	1e-95
CCACATTCCA	TEAD1	1e-43

IFN-γ (+)

Motif	TF	P value
CCTGGAATGC	TEAD4	1e-222
CCACATTCCA	TEAD1	1e-221
GATGAGTCATCC	Jun-AP1	1e-145
CAAACCACAG	RUNX1	1e-67
CAAACCACAAAC	RUNX2	1e-51

Supplementary Figure 5 | PHF8 represses retrotransposon expression in a demethylase-independent manner. a, PHF8 ChIP–seq analysis. Heatmaps (*upper*) and read-count tag density pileup (*lower*) illustrating the density of PHF8 ChIP–seq signal reads. **b,** H3K9me1, H3K9me2, and H3K27me2 ChIP–seq data. Heatmaps illustrating the density of H3K9me1, H3K9me2, and H3K27me2 ChIP–seq signal reads (*upper*) and read-count tag density pileups of H3K9me1, H3K9me2, and H3K27me2 ChIP–seq (*lower*) are shown. **c,** H4K20me1 ChIP–seq analysis. Heatmaps and read-count tag density pileup illustrating the density of H4K20me1 ChIP–seq signal reads (*left*), genomic annotations of H4K20me1 binding peaks (*upper right*) and metagene profiles of H4K20me1 signals along transcripts (*lower right*) are shown. **d,** ChIP–qPCR data showing binding patterns of H3K9me1, H3K9me2, H3K27me2, and H4K20me1 on retrotransposons in the control and *Phf8* KO CT26 cells. **e,** Integrative genomic viewer (IGV) screenshots of aggregated H3K9me3 ChIP–seq signals of transposable element (TE) loci in the control and *Phf8* KO CT26 cells. Values are expressed as mean ± SEM. n = 3 biologically independent samples. **f,** IGV screenshots of aggregated H3K9me3 ChIP–seq signals of *IAPEY3-int* loci (*left*) and its average change of H3K9me3 levels (*right*). n = 2 biological replicates per group. Boxplots denote the medians and the interquartile ranges (IQR). The whiskers of a boxplot are the lowest datum still within 1.5 IQR of the lower quartile and the highest datum still within 1.5 IQR of the upper quartile. Data are presented as the mean ± s.e.m.. **g,** Motif sequences (*left*) and matched transcription factor (TF) with corresponding P value (*right*) from increased ATAC-seq peaks in *Phf8* KO CT26 cells compared with the control cells treated with or without 20 ng/mL IFN-γ for 24 hours. **h,** IGV screenshots of aggregated ATAC–seq signals of selected ISG loci in the control and *Phf8* KO CT26 cells treated with or without IFN-γ.

Supplementary Figure 6
Related to Figure 6

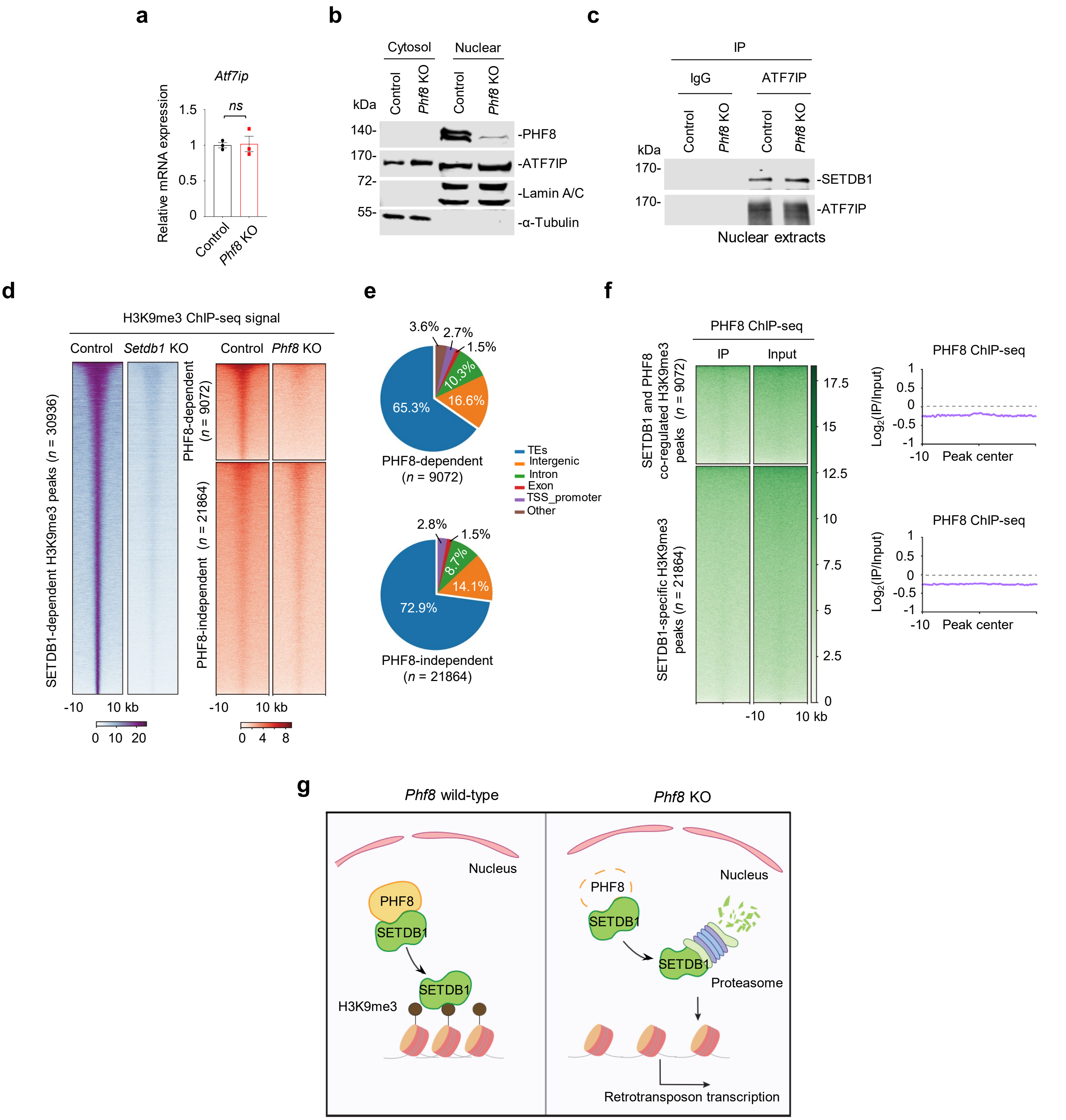

Supplementary Figure 6 | PHF8 and SETDB1 in large part control the same H3K9me3-binding peaks. a, RT-qPCR analysis of *Atf7ip* mRNA expression in the vector control and *Phf8* KO CT26 cells. Values are expressed as mean ± SEM. *n* = 3 biologically independent samples. Unpaired two-sided Student's *t*-test. *ns*, not significant. **b,** Immunoblot analysis for PHF8 and ATF7IP expression in the nuclear and cytoplasmic fractions. Lamin A/C or α-tubulin was used as protein loading controls for the nuclear and cytoplasmic fractions, respectively. **c,** Western blot analysis of ATF7IP immunoprecipitates from nuclear extracts from the vector control and *Phf8* KO CT26 cells. **d**, Heatmap of the H3K9me3 ChIP–seq signals within H3K9me3 peaks in the control and *Setdb1* KO B16 cells (*left*) or control and *Phf8* KO CT26 cells (*right*). **e,** Genomic annotations of PHF8-dependent H3K9me3 binding peaks (*n* = 9072, *upper*) and PHF8-independent H3K9me3 binding peaks (*n* = 21864, *lower*) in CT26 cells. **f,** Heatmap of the PHF8 ChIP–seq signals within SETDB1 and PHF8 co-regulated H3K9me3 binding peaks (*n* = 9072, *upper left*) and SETDB1-specific H3K9me3 binding peaks (*n* = 21864, *lower left*) in CT26 cells. Read-count tag density pileups of PHF8 profiles on SETDB1 and PHF8 co-regulated H3K9me3 binding peaks (*n* = 9072, *upper right*) and SETDB1-specific H3K9me3 binding peaks (*n* = 21864, *lower right*) in CT26 cells. **g,** The working model showing that PHF8 suppresses H3K9me3-modified retrotransposons by sustaining the nuclear stability of SETDB1. The immunoblots in **b** and **c** are representative of three independent experiments. Source data are provided as a Source Data file.

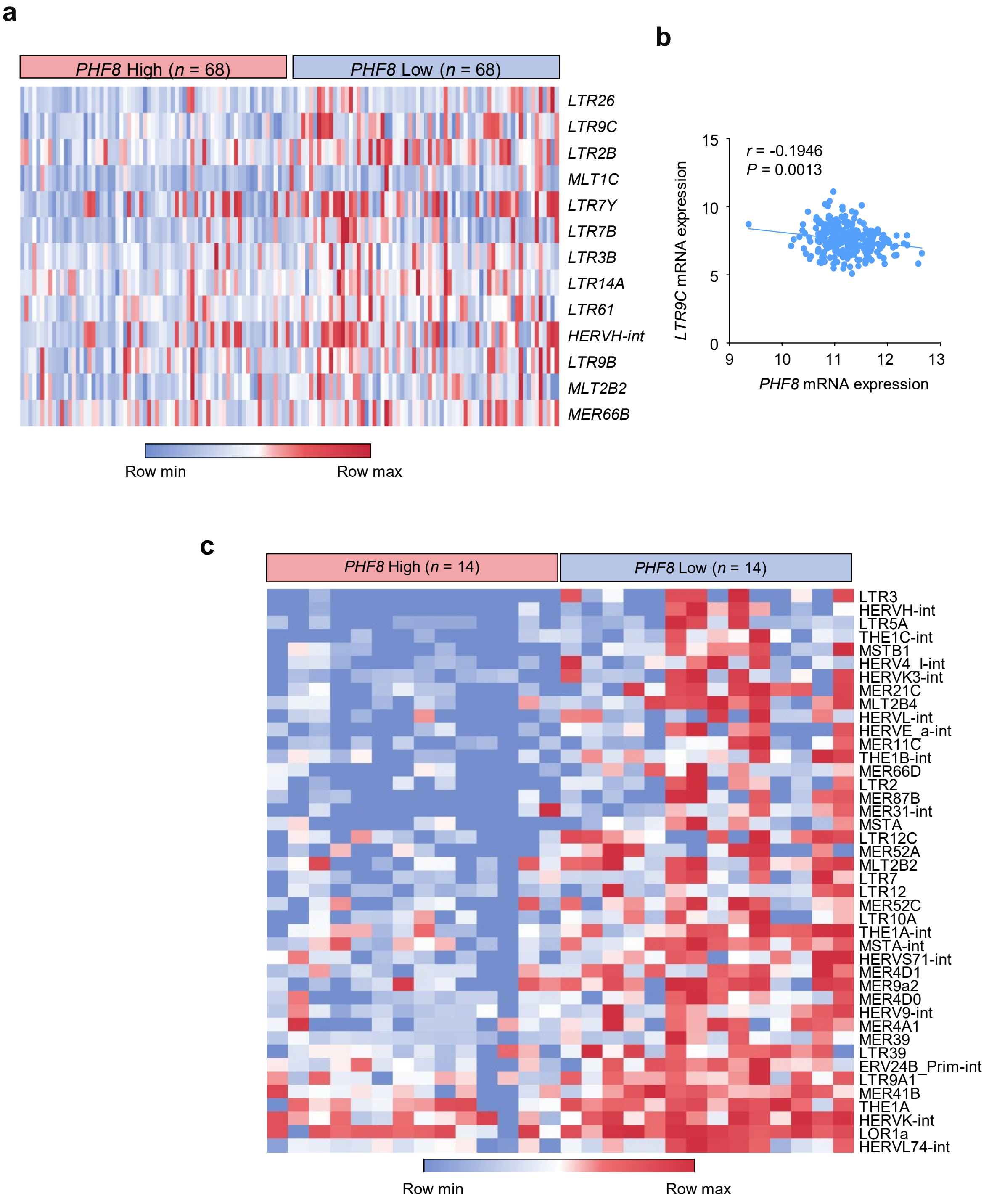

Supplementary Figure 7 | Correlation analysis of human ERV expression with *PHF8* expression in colorectal adenocarcinoma patients and human colorectal cell lines. a, Heatmap showing differential expression ($P < 0.05$) of retrotransposons in *PHF8* high- ($n = 68$) or low-expressed ($n = 68$) colorectal adenocarcinoma patients. Upregulated genes (Log_2 FD > 0, $P < 0.05$) in *PHF8* low-expressed patients compared with *PHF8* high-expressed patients are shown. **b,** *LTR9C* (*left*) and *LTR2B* (*right*) expression were inversely correlated with *PHF8* expression in the colorectal tumor patient cohort. **c,** Heatmap showing differential expression ($P < 0.05$) of retrotransposons in *PHF8* high- ($n = 14$) or low-expressed ($n = 14$) colorectal tumor cell lines. Upregulated genes (Log_2 FD > 0.5, $P < 0.05$) in *PHF8* low-expressed cell lines compared with *PHF8* high-expressed cell lines are shown. *P* value was calculated by unpaired two-sided Student's *t*-test in **a** and **c**.
