# Supplementary material for: Loss of PHF8 induces a viral mimicry response by activating endogenous retrotransposons
Source: Nat Commun. 2023 Jul 15;14:4225. doi: 10.1038/s41467-023-39943-y (PMC10349869; doi:10.1038/s41467-023-39943-y)
Supplement: Supplementary file 3 — Description of Additional Supplementary Files [file 41467_2023_39943_MOESM3_ESM.pdf]

Description of Additional Supplementary Files

Supplementary Data 1. List of differentially expressed genes from strand-specific RNA-seq analysis of *Phf8* KO CT26 cells *vs.* the vector control cells.

Supplementary Data 2. List of differentially expressed genes from strand-specific RNA-seq analysis of *Phf8* KO + *Phf8* CT26 cells *vs. Phf8* KO cells.

Supplementary Data 3. GO analysis of RNA-seq data showing upregulated pathways in *Phf8* KO CT26 cells compared with the vector control cells.

Supplementary Data 4. GO analysis of RNA-seq data showing downregulated pathways in *Phf8* KO + *Phf8* CT26 cells compared with *Phf8* KO cells.

Supplementary Data 5. KEGG analysis of RNA-seq data showing upregulated pathways in *Phf8* KO CT26 cells compared with the vector control cells.

Supplementary Data 6. KEGG analysis of RNA-seq data showing downregulated pathways in *Phf8* KO + *Phf8* CT26 cells compared with *Phf8* KO cells.

Supplementary Data 7. List of differential expressed retroelement loci from strand-specific RNA-seq analysis of *Phf8* KO CT26 cells *vs.* the vector control cells.

Supplementary Data 8. List of differential expressed retroelement loci from strand-specific RNA-seq analysis of *Phf8* KO + *Phf8* CT26 cells *vs. Phf8* KO cells.

Supplementary Data 9. List of sgRNA, shRNA and siRNA sequences used.

Supplementary Data 10. List of primers used for RT-qPCR assays.
