# Supplementary material for: Loss of PHF8 induces a viral mimicry response by activating endogenous retrotransposons
Source: Nat Commun. 2023 Jul 15;14:4225. doi: 10.1038/s41467-023-39943-y (PMC10349869; doi:10.1038/s41467-023-39943-y)
Supplement: Supplementary file 14 — Reporting Summary [file 41467_2023_39943_MOESM14_ESM.pdf]

nature portfolio

Corresponding author(s): Xiufeng Pang, Jiangping He

Last updated by author(s): Jun 24,2023

Reporting Summary

Nature Portfolio wishes to improve the reproducibility of the work that we publish. This form provides structure for consistency and transparency in reporting. For further information on Nature Portfolio policies, see our Editorial Policies and the Editorial Policy Checklist.

Statistics

For all statistical analyses, confirm that the following items are present in the figure legend, table legend, main text, or Methods section.

n/a	Confirmed	
☐	☒	The exact sample size (*n*) for each experimental group/condition, given as a discrete number and unit of measurement
☐	☒	A statement on whether measurements were taken from distinct samples or whether the same sample was measured repeatedly
☐	☒	The statistical test(s) used AND whether they are one- or two-sided<br>*Only common tests should be described solely by name; describe more complex techniques in the Methods section.*
☐	☒	A description of all covariates tested
☐	☒	A description of any assumptions or corrections, such as tests of normality and adjustment for multiple comparisons
☐	☒	A full description of the statistical parameters including central tendency (e.g. means) or other basic estimates (e.g. regression coefficient) AND variation (e.g. standard deviation) or associated estimates of uncertainty (e.g. confidence intervals)
☐	☒	For null hypothesis testing, the test statistic (e.g. *F*, *t*, *r*) with confidence intervals, effect sizes, degrees of freedom and *P* value noted<br>*Give P values as exact values whenever suitable.*
☒	☐	For Bayesian analysis, information on the choice of priors and Markov chain Monte Carlo settings
☒	☐	For hierarchical and complex designs, identification of the appropriate level for tests and full reporting of outcomes
☒	☐	Estimates of effect sizes (e.g. Cohen's *d*, Pearson's *r*), indicating how they were calculated

Our web collection on statistics for biologists contains articles on many of the points above.

Software and code

Policy information about availability of computer code

Data collection	BD FACSDiva (V.8.0.1) was used to collect flow cytometric data.
Data analysis	GraphPad Prism (v8), Microsoft Excel (v15), FlowJo (v10), cBioportal (webtool, no version, http://www.cbioportal.org), OncoDB (webtool, no version, https://oncodb.org), ICBatlas (webtool, no version, http://bioinfo.life.hust.edu.cn/ICBatlas), SAMtools (v.1.9), deepTools (v3.0.2), bedtools (v2.27.1), bowtie2 (v2.3.4), STAR aligner (v2.5.4b), DESeq2 (v1.20.0), Picard (https://broadinstitute.github.io/picard/), MACS2 (v.2.1.2), RepeatMasker (http://www.repeatmasker.org).

For manuscripts utilizing custom algorithms or software that are central to the research but not yet described in published literature, software must be made available to editors and reviewers. We strongly encourage code deposition in a community repository (e.g. GitHub). See the Nature Portfolio guidelines for submitting code & software for further information.

Data

Policy information about availability of data

All manuscripts must include a data availability statement. This statement should provide the following information, where applicable:

- Accession codes, unique identifiers, or web links for publicly available datasets
- A description of any restrictions on data availability
- For clinical datasets or third party data, please ensure that the statement adheres to our policy

All genomic sequencing data that support the findings generated in this study have been deposited in the Gene Expression Omnibus database under the accession

GSE212779 (https://www.ncbi.nlm.nih.gov/gds/?term=GSE212779) and GSE211526 (https://www.ncbi.nlm.nih.gov/gds/?term=GSE211526). The publicly available data on gene expression of colorectal adenocarcinoma were obtained from cBioportal 75 (http://www.cbioportal.org). The publicly available data on overall survival of colorectal adenocarcinoma were obtained from OncoDB 70 (https://oncodb.org). The publicly available data on PHF8 gene expression and immunotherapy response were obtained from ICBatlas 71 (http://bioinfo.life.hust.edu.cn/ICBatlas). The publicly available data on ERV expression were obtained from Mendeley Data 40 (https://data.mendeley.com/datasets/c7r7dw9p42/1). The publicly available data of H3K9me3 ChIP-seq in the control and SETDB1 KO B16 melanoma cells were downloaded and reanalyzed from the Gene Expression Omnibus database under the accession GSE155972 9 (https://www.ncbi.nlm.nih.gov/gds/?term=GSE155972). The remaining data are available with the Article, Supplementary Information or Source data file. Source data are provided with this paper.

Human research participants

Policy information about studies involving human research participants and Sex and Gender in Research.

Reporting on sex and gender	None
Population characteristics	None
Recruitment	None
Ethics oversight	None

Note that full information on the approval of the study protocol must also be provided in the manuscript.

Field-specific reporting

Please select the one below that is the best fit for your research. If you are not sure, read the appropriate sections before making your selection.

☒ Life sciences ☐ Behavioural & social sciences ☐ Ecological, evolutionary & environmental sciences

For a reference copy of the document with all sections, see nature.com/documents/nr-reporting-summary-flat.pdf

Life sciences study design

All studies must disclose on these points even when the disclosure is negative.

Sample size	Experimental samples sizes were selected according to commonly accepted standards for in vitro and in vivo validation of tumor inoculations and immune checkpoint therapy and to achieve statistical significance. Similarly, group sizes for in vitro experiments were selected on the basis of prior knowledge of variation.
Data exclusions	None
Replication	Replicates were used in all experiments as indicated in text, figure legends and methods. Animal experiments have been repeated as indicated in the figure legends. All other experiments have been repeated at least twice with consonant results.
Randomization	For in vivo experiments, tumor-bearing mice were randomly assigned to experimental groups prior to treatments. For in vitro experiments, randomization was done wherever possible.
Blinding	Blinding was not done due to the requirements for case labeling and staffing needs, as knowledge of the grouping information was essential for the staff to conduct the studies.

Reporting for specific materials, systems and methods

We require information from authors about some types of materials, experimental systems and methods used in many studies. Here, indicate whether each material, system or method listed is relevant to your study. If you are not sure if a list item applies to your research, read the appropriate section before selecting a response.

Materials & experimental systems

n/a	Involved in the study	
☐	☒	Antibodies
☐	☒	Eukaryotic cell lines
☒	☐	Palaeontology and archaeology
☐	☒	Animals and other organisms
☒	☐	Clinical data
☒	☐	Dual use research of concern

Methods

n/a	Involved in the study	
☐	☒	ChIP-seq
☐	☒	Flow cytometry
☒	☐	MRI-based neuroimaging

Antibodies

Antibodies used

For western blots: anti-PHF8, abcam, ab280887, 1:1,000; anti-RIG-I, CST, #3743, 1:1,000; anti-MDA5, CST, #5321, 1:1,000; anti-MAVS, CST, #4983, 1:1,000; anti-cGAS, CST, #31659, 1:1,000; anti-p-TBK1, CST, #5483, 1:1,000; anti-TBK1, CST, #3504, 1:1,000; anti-p-IRF3, CST, #4947, 1:1,000; anti-IRF3, CST, #4302, 1:1,000; anti-SUV39H1, CST, #8729, 1:1,000; anti-α-Tubulin, CST, #2125, 1:1,000; anti-Lamin A/C, CST, #4777, 1:1,000; anti-p-STAT1, CST, #9167, 1:1,000; anti-STAT1, CST, #9172, 1:1,000; anti-GAPDH, Abways, AB0036, 1:1,000; anti-SETDB1, Proteintech, 11231-1-AP, 1:1,000; anti-ATF7IP, Santa Cruz Biotechnology, sc-166753, 1:1,000.

For ChIP-seqs: anti-H3K9me1, abcam, ab9045; anti-H3K9me2, abcam, ab176882; anti-H3K9me3, CST, #13939; anti-H3K27me2, abcam, ab24684; anti-H4K20me1, abcam, ab177188; anti-PHF8, abcam, ab280887.

For flow cytometry: Zombie AquaTM fixable viability dye, BioLegend, 423101; anti-CD45-PerCP/Cyanine5.5, BioLegend, clone 30-F11, 103131; anti-CD8a-APC, BioLegend, clone 53-6.7, 100711; anti-CD62L-PE, BioLegend, clone MEL-14, 144407; anti-CD44-PE/Cyanine7, BioLegend, clone IM7, 103030.

For immunofluorescence: anti-dsRNA monoclonal antibody J2, SCICONS, 10010200; anti-dsDNA monoclonal antibody, Merck, MAB1293; anti-SETDB1, Proteintech, 11231-1-AP; anti-CD8a, Servicebio, GB13429; anti-IFN-γ, Servicebio ,GB11107-1; anti-GZMB, Affinity, AF0175.

For immunohistochemistry: anti-PHF8, Servicebio, GB114477.

For immunotherapy treatments: anti-PD-1 antibodies, BioLegend, clone RMP1-14, 114101.

Validation

All antibodies used in this study are commercially available and have been validated by manufacturer. Any validation statements are available on the manufacturer's website.The RRID of each antibodies is also provided here:

The following antibodies were purchased from abcam:
anti-H3K9me1, ab9045, RRID: AB_306963, 1:1000;
anti-H3K9me2, ab176882, RRID: AB_2895140, 1:1000;
anti-H3K27me2, ab24684, RRID: AB_448222, 1:1000.

The following antibodies were purchased from Cell Signaling Technology:
anti-RIG-I, #3743, RRID: AB_2269233, 1:1,000;
anti-MDA5, #5321, RRID: AB_10694490, 1:1,000;
anti-MAVS, #4983, RRID: AB_823566, 1:1,000;
anti-cGAS, #31659, RRID: AB_2799008, 1:1,000;
anti-p-TBK1, #5483, RRID: AB_10693472, 1:1,000;
anti-TBK1, #3504, RRID: AB_2255663, 1:1,000;
anti-p-IRF3, #4947, RRID: AB_823547, 1:1,000;
anti-IRF3, #4302, RRID: AB_1904036, 1:1,000;
anti-SUV39H1, #8729, RRID: AB_10829612, 1:1,000;
anti-α-Tubulin, #2125, RRID: AB_2619646, 1:1,000;
anti-Lamin A/C, #4777, RRID: AB_10545756, 1:1,000;
anti-STAT1, #9172, RRID: AB_2198300, 1:1,000;
anti-H3K9me3, #13939, RRID: AB_2798355, 1:1,000.

The following antibodies were purchased from Abways:
anti-GAPDH, AB0036, 1:1,000.

The following antibodies were purchased from Proteintech:
anti-SETDB1, 11231-1-AP, RRID: AB_2186069, 1:1,000.

The following antibodies were purchased from Santa Cruz Biotechnology:
anti-ATF7IP, sc-166753, 1:1,000.

The following antibodies were purchased from BioLegend:
anti-CD45-PerCP/Cyanine5.5, clone 30-F11, 10313, RRID: AB_893344;
anti-CD8a-APC, clone 53-6.7, 100711, RRID: AB_312750;
anti-CD62L-PE, clone MEL-14, 144407, RRID:AB_313094;
anti-CD44-PE/Cyanine7, clone IM7, 103030, RRID: AB_830787;
anti-PD-1 antibodies, clone RMP1-14, 114101, RRID:AB_313572.

The following antibodies were purchased from SCICONS:
anti-dsRNA monoclonal antibody J2, 10010200, RRID: AB_2651015.

The following antibodies were purchased from Merck:
anti-dsDNA monoclonal antibody, MAB1293, RRID:AB_94097.

The following antibodies were purchased from Servicebio:
anti-CD8a, GB13429, RRID: AB_290418.

The following antibodies were purchased from Affinity:

anti-GZMB, AF0175, RRID: AB_2833368.

Eukaryotic cell lines

Policy information about cell lines and Sex and Gender in Research

Cell line source(s)	CT26 (RRID:CVCL_7254), MC38 (RRID:CVCL_0A68), 4T1 (RRID:CVCL_0125), 293T (CRL-11268), HT-29 (RRID:CVCL_0320), and LoVo (RRID:CVCL_0399) cells were purchased from the American Type Culture Collection. KPC cells were isolated from mouse PDAC tumors driven by mutant Kras and mutant Trp53 and gifted from Dr. Zhigang Zhang (Shanghai Jiao Tong University, Shanghai, China).
Authentication	All the cell lines were reported previously and obtained from ATCC, but were not authenticated further.
Mycoplasma contamination	All the cell lines were authenticated by the short tandem repeat method and tested negative for mycoplasma.
Commonly misidentified lines (See ICLAC register)	None

Animals and other research organisms

Policy information about studies involving animals; ARRIVE guidelines recommended for reporting animal research, and Sex and Gender in Research

Laboratory animals	Six-8 week-old C57BL/6 mice, Balb/c mice, and nude mice were purchased from the National Rodent Laboratory Animal Resources (Shanghai, China). Six-8 week-old RAG2-/- mice (C57BL/6 background) were gifted by Dr. Bing Du (East China Normal University, Shanghai, China).
Wild animals	We did not use any wild animals.
Reporting on sex	To avoid potential impacts of physiological cycle of female mice on experimental results, we chose male BALB/c, C57BL/6, nude and RAG2-/- mice for our in vivo animal studies.
Field-collected samples	This study did not involved field-collected samples.
Ethics oversight	The mouse experiment procedures were approved by the Institutional Animal Care and Use Committee of East China Normal University and conducted in accordance with the guidelines (protocol number AR2021-265).

Note that full information on the approval of the study protocol must also be provided in the manuscript.

ChIP-seq

Data deposition

☒ Confirm that both raw and final processed data have been deposited in a public database such as GEO.

☒ Confirm that you have deposited or provided access to graph files (e.g. BED files) for the called peaks.

Data access links<br>*May remain private before publication.*	'https://www.ncbi.nlm.nih.gov/geo/query/acc.cgi?acc=GSE212779' and 'https://www.ncbi.nlm.nih.gov/geo/query/acc.cgi?acc=GSE211526'
Files in database submission	Superseries GEO Acession: GSE212779 and GSE211526.<br>Composed of the following sub-series and files:<br><br>RNA-seq raw fastq files (GSE211474):<br>GSM6473203 CT26 sh con_1. fastq.gz<br>GSM6473204 CT26 sh con_2. fastq.gz<br>GSM6473205 CT26 sh con_3. fastq.gz<br>GSM6473206 CT26 sh con_4. fastq.gz<br>GSM6473207 CT26 sh con_5. fastq.gz<br>GSM6473208 CT26 sh con_6. fastq.gz<br>GSM6473209 CT26 sh con_7. fastq.gz<br>GSM6473210 CT26 sh con_8. fastq.gz<br>GSM6473211 CT26 sh1_1. fastq.gz<br>GSM6473212 CT26 sh1_2. fastq.gz<br>GSM6473213 CT26 sh1_3. fastq.gz<br>GSM6473214 CT26 sh1_4. fastq.gz

GSM6473215 CT26 sh1_5. fastq.gz
GSM6473216 CT26 sh1_6. fastq.gz
GSM6473217 CT26 sh1_7. fastq.gz
GSM6473218 CT26 sh1_8. fastq.gz
GSM6473219 CT26 sh2_1. fastq.gz
GSM6473220 CT26 sh2_2. fastq.gz
GSM6473221 CT26 sh2_3. fastq.gz
GSM6473222 CT26 sh2_4. fastq.gz
GSM6473223 CT26 sh2_5. fastq.gz
GSM6473224 CT26 sh2_6. fastq.gz
GSM6473225 CT26 sh2_7. fastq.gz
GSM6473226 CT26 sh2_8. fastq.gz
GSM6473227 PANC28 sgcon_1. fastq.gz
GSM6473228 PANC28 sgcon_2. fastq.gz
GSM6473229 PANC28 sgcon_3. fastq.gz
GSM6473230 PANC28 sgPHF8_1. fastq.gz
GSM6473231 PANC28 sgPHF8_2. fastq.gz
GSM6473232 PANC28 sgPHF8_3. fastq.gz
GSM6473233 CT26 sg con_1. fastq.gz
GSM6473234 CT26 sg con_2. fastq.gz
GSM6473235 CT26 sg con_3. fastq.gz
GSM6473236 CT26 sgPhf8_1. fastq.gz
GSM6473237 CT26 sgPhf8_2. fastq.gz
GSM6473238 CT26 sgPhf8_3. fastq.gz
GSM6473239 CT26 sgPhf8+Phf8_1. fastq.gz
GSM6473240 CT26 sgPhf8+Phf8_2. fastq.gz
GSM6473241 CT26 sgPhf8+Phf8_3. fastq.gz

RNA-seq processed files (GSE211474):
GSE211474_CT26_cell_processed_datafiles_DESeq2.normalized.txt
GSE211474_CT26_tumors_processed_data_files_gene_old.fpkm.txt
GSE211474_PANC28_processed_data_files_gene_old.fpkm.txt

ChIP-seq raw fastq files (GSE211524):
GSM6475131 CT26 con1_H3K9me1. fastq.gz
GSM6475132 CT26 con2_H3K9me1. fastq.gz
GSM6475133 CT26 Phf8 KO1_H3K9me1. fastq.gz
GSM6475134 CT26 Phf8 KO2_H3K9me1. fastq.gz
GSM6475135 CT26 con1_H3K9me2. fastq.gz
GSM6475136 CT26 con2_H3K9me2. fastq.gz
GSM6475137 CT26 Phf8 KO1_H3K9me2. fastq.gz
GSM6475138 CT26 Phf8 KO2_H3K9me2. fastq.gz
GSM6475139 CT26 con1_H3K9me3. fastq.gz
GSM6475140 CT26 con2_H3K9me3. fastq.gz
GSM6475141 CT26 Phf8 KO1_H3K9me3. fastq.gz
GSM6475142 CT26 Phf8 KO2_H3K9me3. fastq.gz
GSM6475143 CT26 con1_H3K27me2. fastq.gz
GSM6475144 CT26 con2_H3K27me2. fastq.gz
GSM6475145 CT26 Phf8 KO1_H3K27me2. fastq.gz
GSM6475146 CT26 Phf8 KO2_H3K27me2. fastq.gz
GSM6475147 CT26 con1_H4K20me1. fastq.gz
GSM6475148 CT26 con2_H4K20me1. fastq.gz
GSM6475149 CT26 Phf8 KO1_H4K20me1. fastq.gz
GSM6475150 CT26 Phf8 KO2_H4K20me1. fastq.gz
GSM6475151 CT26 con1_input. fastq.gz
GSM6475152 CT26 con2_input. fastq.gz
GSM6475153 CT26 Phf8 KO1_ input. fastq.gz
GSM6475154 CT26 Phf8 KO2_ input. fastq.gz

ChIP-seq processed files (GSE211524):
CT26 con1_H3K9me1.bw
CT26 con2_H3K9me1.bw
CT26 Phf8 KO1_H3K9me1.bw
CT26 Phf8 KO2_H3K9me1.bw
CT26 con1_H3K9me2.bw
CT26 con2_H3K9me2.bw
CT26 Phf8 KO1_H3K9me2.bw
CT26 Phf8 KO2_H3K9me2.bw
CT26 con1_H3K9me3.bw
CT26 con2_H3K9me3.bw
CT26 Phf8 KO1_H3K9me3.bw
CT26 Phf8 KO2_H3K9me3.bw
CT26 con1_H3K27me2.bw
CT26 con2_H3K27me2.bw
CT26 Phf8 KO1_H3K27me2.bw
CT26 Phf8 KO2_H3K27me2.bw

CT26 con1_H4K20me1.bw
CT26 con2_H4K20me1.bw
CT26 Phf8 KO1_H4K20me1.bw
CT26 Phf8 KO2_H4K20me1.bw
CT26 con1_input.bw
CT26 con2_input.bw
CT26 Phf8 KO1_ input.bw
CT26 Phf8 KO2_ input.bw

ChIP-seq raw fastq files (GSE212778):
GSM6547601 CT26 con1, PHF8. fastq.gz
GSM6547602 CT26 con2, PHF8. fastq.gz
GSM6547603 CT26 Phf8 KO1, PHF8. fastq.gz
GSM6547604 CT26 Phf8 KO2, PHF8. fastq.gz
GSM6547605 CT26 con1, INPUT. fastq.gz
GSM6547606 CT26 con2, INPUT. fastq.gz
GSM6547607 CT26 Phf8 KO1, INPUT. fastq.gz
GSM6547608 CT26 Phf8 KO2, INPUT. fastq.gz

ChIP-seq processed files (GSE212778):
CT26 con1_PHF8.bw
CT26 con2_PHF8.bw
CT26 Phf8 KO1_PHF8.bw
CT26 Phf8 KO2_PHF8.bw
CT26 con1_INPUT.bw
CT26 con2_INPUT.bw
CT26 Phf8 KO1_INPUT.bw
CT26 Phf8 KO2_INPUT.bw

ATAC-seq raw fastq files (GSE211525):
GSM6475155 ATAC_CT26_con_1
GSM6475156 ATAC_CT26_con_2
GSM6475157 ATAC_CT26_Phf8 KO_1
GSM6475158 ATAC_CT26_Phf8 KO_2
GSM6475159 ATAC_CT26_IFNcon_1
GSM6475160 ATAC_CT26_IFNcon_2
GSM6475161 ATAC_CT26_IFNPhf8 KO_1
GSM6475162 ATAC_CT26_IFNPhf8 KO_2

ATAC-seq processed files (GSE211525):
ATAC_CT26_con_1.bw
ATAC_CT26_con_2.bw
ATAC_CT26_Phf8 KO_1.bw
ATAC_CT26_Phf8 KO_2.bw
ATAC_CT26_IFNcon_1.bw
ATAC_CT26_IFNcon_2.bw
ATAC_CT26_IFNPhf8 KO_1.bw
ATAC_CT26_IFNPhf8 KO_2.bw

Genome browser session
(e.g. UCSC)

http://genome.ucsc.edu/cgi-bin/hgTracks?db=mm10&lastVirtModeType=default&lastVirtModeExtraState=&virtModeType=default&virtMode=0&nonVirtPosition=&position=chr10%3A7228828%2D7687847&hgsid=879674613_myha9YKuA32HOgjtBPP8VFlTx8bb

Methodology

Replicates

CT26 cells ChIP-seq: 2 replicate of each of the following: CT26 con_H3K9me1, CT26 Phf8 KO_H3K9me1, CT26 con_H3K9me2, CT26 Phf8 KO_H3K9me2, CT26 con_H3K9me3, CT26 Phf8 KO_H3K9me3, CT26 con_H3K27me2, CT26 Phf8 KO_H3K27me2, CT26 con_H4K20me1, CT26 Phf8 KO_H4K20me1, CT26 con_input, CT26 Phf8 KO_ input, CT26 con_PHF8, CT26 Phf8 KO_PHF8, CT26 con_INPUT, CT26 Phf8 KO_INPUT;
CT26 tumor tissues RNA-seq: 8 replicate of each of the following: CT26 sh con, CT26 sh1, CT26 sh2;
CT26 cells RNA-seq: 3 replicate of each of the following: CT26 sg con, CT26 sgPhf8, CT26 sgPhf8+Phf8;
CT26 cells ATAC-seq: 2 replicate of each of the following: CT26_con, CT26_Phf8 KO, CT26_IFNcon, CT26_IFNPhf8 KO.

Sequencing depth

Approximately 30-80 million paired-end reads per sample with 150bp total read length.

Antibodies

For CT26 ChIP-seqs: anti-H3K9me1(ab9045, abcam), anti-H3K9me2 (ab176882, abcam), anti-H3K9me3 (13939, CST), anti-H3K27me2 (ab24684, abcam), anti-H4K20me1 (ab177188, abcam), anti-PHF8 (ab280887, abcam).
No Antibodies for RNA seq.

Peak calling parameters

ChIP-seq and ATAC-seq: ChIP-seq and ATAC-seq reads were aligned to the mouse mm10 genome using bowtie2 with the options '-p 20 –very-sensitive –end-to-end –no-unal–no-mixed -X 2000'. ChIP-seq enriched peaks were called by MACS2 (v.2.1.2) and SICER2, and the common peaks were merged using bedtools and used for subsequent analysis. ATAC-seq peaks were called using DFilter (with the settings: -bs = 100 –ks = 60 –refine).

	RNA-seq: Reads were aligned to the reference transcriptome using RSEM and bowtie and the index was built by RSEM with the mouse genome, mm10, and Enemble gene annotation track v.74. For TE analysis, the reads were mapped to the mouse genome (mm10) using the STAR aligner and the counts for each gene or TE family were counted using scTE. DESeq2 was used for data normalization and differential expression analysis.
Data quality	Reads were trimmed using Trimmomatic or Trim galore.
Software	SAMtools (v.1.9), deepTools v3.0.2, bedtools v2.27.1, bowtie2 v2.3.4, STAR 2.5.4b , TEtranscripts vNA, MEME v4.12.0, glbase/glbase3 vNA, DESeq2 v1.20.0.

Flow Cytometry

Plots

Confirm that:

☒ The axis labels state the marker and fluorochrome used (e.g. CD4-FITC).

☒ The axis scales are clearly visible. Include numbers along axes only for bottom left plot of group (a 'group' is an analysis of identical markers).

☒ All plots are contour plots with outliers or pseudocolor plots.

☒ A numerical value for number of cells or percentage (with statistics) is provided.

Methodology

Sample preparation	Tumor cells were firstly prepared as single-cell suspensions. To omit dead cells during analysis, cells were stained with viability stain (423101, Zombie AquaTM fixable viability dye, BioLegend) before assay. For MHC class I detection, tumor cells per condition were stained with FITC anti-mouse H-2Kb/H-2Db antibody (114605, BioLegend) diluted in PBS plus 2% FBS for 30 minutes on ice. For the intracellular dsRNA detection, tumor cells were permeabilized, blocked with 0.1% Triton X-100 in FACS buffer for 15 minutes at room temperature and then stained with the intracellular dsRNA detection using J2 antibody (10010200, SCICONS).
Instrument	BD LSR II
Software	BD FACSDiva(V.8.0.1) software used for data collection, FlowJo (v10) software used for analysis
Cell population abundance	Cellular size and viability stain (423101, Zombie AquaTM fixable viability dye, BioLegend) were used to exclude dead cells. Positive populations were defined using unstained cells as references.
Gating strategy	All gates were set based on single-stained compensation controls.

☒ Tick this box to confirm that a figure exemplifying the gating strategy is provided in the Supplementary Information.
